# Systemic stem cell treatment rescues injured motoneurons by reducing L-selectin expression on leukocytes

**DOI:** 10.1186/s13287-025-04283-9

**Published:** 2025-05-13

**Authors:** Tamás Bellák, Zoltán Fekécs, Dénes Török, Rebeka Kristóf, Omoikhoje Esezoobo, Annamária Marton, Csaba Vizler, Antal Nógrádi, Krisztián Pajer

**Affiliations:** 1 Department of Anatomy, Histology and Embryology, Albert Szent-Györgyi Medical School, University of Szeged, Szeged, Hungary; 2 National Biotechnology Laboratory, Institute of Genetics, Biological Research Center, Szeged, Hungary; 3 Present address: Galway University Hospitals, Galway, Ireland

**Keywords:** Motoneuron injury, Stem cells, Fucoidan, Neuroinflammation, Regeneration, Functional recovery

## Abstract

**Background:**

Avulsion injury results in motoneuron death due to the increased cytotoxicity developing after the injury. We have earlier shown that intraspinally grafted immortalized NE-4C neuroectodermal stem cells derived from 9-day old mouse forebrain vesicles produced a secretome, which induced decreased microglia/macrophage reaction, and promoted the neuroprotection and regeneration following avulsion injury. Here we intended to prove the motoneuron rescuing effect of intravenously grafted NE-4C stem cells and reveal the mechanism of action used by the grafted cells.

**Methods:**

In our experimental model the left lumbar 4 (L4) ventral root of the spinal cord was avulsed and then reimplanted into the L4 spinal segment. Treated animals received various doses of NE-4C stem cells intravenously and the survival and regeneration of the affected motoneurons was checked by morphological and functional analysis. The molecular changes within the treated cord were followed by the ELISA Proteome Profiler rat cytokine array and qPCR analysis. To mimic the effect of stem cells fucoidan treatment (a specific selectin inhibitor, 50 and 100 mg/kg bw) was applied for two weeks intraperitoneally.

**Results:**

High doses of intravenous stem cell treatment (4 × 10^5^ and 1 × 10^6^ cells) induced the reinnervation of the reimplanted ventral root by surviving injured motoneurons (up to 38% of the total L4 pool). Proteome Profiler analysis showed that systemic stem cell treatment downregulated the level of L-selectin, that promotes leukocyte rolling on vascular endothelium. Both systemic stem cell and fucoidan treatment reduced macrophage and microglial densities in the affected spinal segment and administration of fucoidan downregulated inflammatory cytokine and inflammasome levels along with improved morphological and functional reinnervation.

**Conclusions:**

Blocking L-selectin, similarly to systemic NE-4C stem cell treatment decreases the neuroinflammation in the injured spinal cord segment after ventral root avulsion and induces significant motoneuron survival and functional reinnervation of the denervated hind limb muscles.

## Background

Ventral root avulsion injury induces a devastating damage to the affected motoneurons, whose axons have been torn out, resulting in the death of the vast majority of these cells [1–5]. The loss of the large number of the motoneurons leads to severe functional motor deficit of the affected limb. The avulsed ventral root can be reimplanted into the affected segment but only few surviving motoneurons are able to reinnervate the reimplanted root and contribute to functional reinnervation of denervated muscles [2, 4–6]. Following an avulsion injury, strong microglia/macrophage reaction can be observed throughout the affected spinal segment that leads to number of inflammatory cascades with negative effects on the ventral horn [2]. The activated microglia/macrophages reportedly produce inflammatory cytokines knowing to be deleterious to the highly vulnerable damaged motoneurons [7].

Numerous studies have provided evidence that neural stem cells are promising candidates for cell therapy in ventral root avulsion injuries [1–3, 8, 9]. In our earlier studies, we have also shown that intraspinally grafted immortalized NE-4C neuroectodermal stem cells derived from 9-day old mouse forebrain vesicles [10] produced a set of factors (stem cell secretome) which induced decreased microglia/macrophage reaction, and promoted the neuroprotection and regeneration following avulsion injury [2, 11]. It appeared evident that the number of grafted stem cell showed a linear correlation with the morphological and function improvement, reaching a plateau effect with transplantation of 300.000 stem cells. The question raised whether intravenous administration of NE-4C stem cells, similarly to intraspinal grafting is able to rescue the injured motoneurons otherwise destined to die. Even if intravenous administration effectively rescues damaged motoneurons, it still appears unlikely, that systemic application of the NE-4C cells results in the settling of satisfactorily high numbers of stem cells within the spinal cord or alternatively circulating stem cells would produce high amounts of their secretome composed of cytokines into the systemic circulation without side effects, and these cytokines would produce a therapeutic effect in the injured cord. Therefore, it is hypothesized that other, not stem cell secretome-based mechanism of action is responsible for the motoneuron rescuing effect.

In this study we made attempts to demonstrate that immediate intravenous administration of NE-4C stem cells following lumbar 4 (L4) ventral root avulsion and reimplantation was able to rescue the majority of injured motoneurons and these motoneurons were able to reinnervate the denervated hind limb muscles. The aim of this study was twofold. First, we wanted to prove the motoneuron rescuing effect of intravenously grafted NE-4C stem cells and second to reveal the mechanism of action used by the grafted cells.

## Methods

### Project overview

All together 95 Sprague-Dawley (SD) female animals were used (body weight: 220–250 g.). The individual rat was considered as an experimental unit. The following experimental groups and experimental time line have been set up:

intact group: animals without any intervention (*n* = 4).

AVR group: L4 ventral root was avulsed and reimplanted without stem cell treatment (*n* = 23).

AVR + 2 × 10^5^ NE-TR-4C group: L4 ventral root was avulsed and reimplanted and 2 × 10^5^ NE-TR-4C cells were injected into the tail vein. (*n* = 14)

AVR + 4 × 10^5^ NE-TR-4C: L4 ventral root was avulsed and reimplanted and 4 × 10^5^ NE-TR-4C cells were injected into the tail vein. (*n* = 14)

AVR + 1 × 10^6^ NE-TR-4C: L4 ventral root was avulsed and reimplanted and 1 × 10^6^ NE-TR-4C cells were injected into the tail vein. (*n* = 20)

AVR + 50 mg Fucoidan: L4 ventral root was avulsed and reimplanted and 50 mg Fucoidan was administrated intraperitoneally daily for 1 week and every second day for the next 1 week (*n* = 10).

AVR + 100 mg Fucoidan: L4 ventral root was avulsed and reimplanted and 100 mg Fucoidan was administrated intraperitoneally daily for 1 week and every second day for the next 1 week (*n* = 10).

All the rats were simultaneously randomized to the treatment groups. A cage was randomly selected from the pool of all cages for each group. We reduced the required number of rats for our experiments in order to ascertain statistical significance. Sample size was estimated using PS: Power and Sample Size Calculation (version 3.1.6; W.D. Dupont & W.D. Plummer) program.

Animals were given postoperative analgesia and saline (0.9%; 5 ml) to prevent dehydration and received meloxicam (Metacam; 0,5 mg/kg body weight, Boehringer Ingelheim Vetmedica). Then they were allowed to recover and housed in standard rat cages at a controlled room temperature. Rats were observed daily after surgery to detect any sign of infection. Animals were given food and water ad libitum. We provide environmental enrichment for rodents by using paper cylinders and placing pieces of chewing wood. We excluded the rats that were inconsistently injured.

After various survival times (1, 3, 7 and 84 days) animals were euthanasia with an overdose of ketamine-xylazine (ketamine hydrochloride [Ketasol, Dr. E. Graeub, Bern, Switzerland, 110 mg/kg body weight]; xylazine [Rompun, Byer, Vienna, Austria, 12 mg/kg body weight]) and perfused transcardially with saline containing heparin followed by 4% paraformaldehyde (PFA) in 0.1 mol/l phosphate buffer (pH 7.4).

All the experiments were conducted in a blinded fashion. The first investigator (TB) was responsible for conducting spinal cord injury and stem cell transplantation or fucoidan treatment. The second investigator carried out functional analysis (ZF and DT), whereas a third investigator (RK, OE, AM and CSV) performed data collection and tissue analysis. Finally, a fourth investigator (KP) assessed, analysed, and interpreted overall data. The corresponding author (AN) was the only person aware of the treatment group allocation. All experiments were designed and reported in accordance with the Animal Research: Reporting of In Vivo Experiments (ARRIVE) guidelines.

### Maintenance of NE-TR-4C stem cells

The NE-4C clonal neuroectodermal stem cells (ATCC No. CRL-2926) were isolated from forebrain vesicles of 9-day old embryos of transgenic mice lacking the tumor suppressor gene p53 [10]. The original NE-4C cell line was modified to produce enhanced Tomato Red (TR) protein (further: NE-TR-4C cells). NE-TR-4C cells were maintained on nuncloned petri dishes (Thermo Fisher Scientific, Waltham, USA) in High glucose Dulbecco Modified Essential Medium (H-DMEM, Sigma-Aldrich, St. Louis, USA) supplemented with 10% fetal calf serum (FCS, Thermo Fisher Scientific, Waltham, USA) at 37 °C and 5% CO_2_.

### Ventral root avulsion–reimplantation and transplantation of NE-TR-4C stem cells

All the operations were carried out under deep ketamine-xylazine anaesthesia (ketamine hydrochloride [Ketasol, Dr. E. Graeub, Bern, Switzerland, 110 mg/kg body weight]; xylazine [Rompun, Byer, Vienna, Austria, 12 mg/kg body weight]) with sterile precautions. To maintain the body temperature at 37.0 ± 0.5 °C, the rats were kept on a heating pad (Supertech Ltd, Pécs, Hungary) during the surgery. Laminectomy was performed at the vertebral level of T13–L1, the dura was opened and the left L4 ventral root was identified and pulled out, leaving the dorsal roots intact. The cut end of the ventral root was then inserted into the ventrolateral part of the spinal cord and various numbers of NE-TR-4C stem cells were injected into the tail vein (AVR + 2 × 10^5^ NE-TR-4C, AVR + 4 × 10^5^ NE-TR-4C, AVR + 1 × 10^6^ NE-TR-4C groups). The spinal cord was covered with the remaining dura, the wound was closed and the animals were allowed to recover. In the control experiments (AVR group), the left L4 ventral root was avulsed and reimplanted without NE-TR-4C stem cells.

### Fucoidan treatment

Fucoidan (from Fucus vesiculosus) was purchased from Sigma-Aldrich (F8190-500MG, Sigma-Aldrich, St. Louis, USA) and dissolved in distilled water as a stock solution. Animals were treated with fucoidan (50 and 100 mg/kg) for 2 weeks. Fucoidan treatment started immediately on the day of surgery (*n* = 10 in each group). The drug was injected intraperitoneally daily for 1 week and every second day for the next 1 week. Three animals remained untreated. This treatment protocol was based on the successful fucoidan treatment described in earlier papers [12].

### Retrograde labeling

Three months after the surgery animals were deeply anesthetized as described above. On the operated side the ventral ramus of the left L4 spinal nerve was cut and the proximal stump of the nerve was covered with Fast Blue crystals (Chemimart GmbH, Berlin, Germany). Five days after the application of the fluorescent dye, the animals were euthanasia with an overdose of ketamine and xylazine (ketamine hydrochloride [Ketasol, Dr. E. Graeub, Bern, Switzerland, 110 mg/kg body weight]; xylazine [Rompun, Byer, Vienna, Austria, 12 mg/kg body weight]) and perfused transcardially with physiological saline (0,9% NaCl) containing heparin followed by 4% paraformaldehyde (PFA, Sigma-Aldrich) in 0.1 M phosphate buffer (pH: 7.4) The spinal cord of the animals was removed and placed into 4% buffered PFA for overnight. The fixed tissues were cryoprotected in 30% sucrose in PBS containing 0.01% sodium-azide at 4 °C until being embedded in Tissue-Tek O.C.T. Compound (Sakura Finetek USA, Torrance, USA). Parallel or serial transverse (25-µm-thick) sections were cut and mounted onto gelatin-coated glass slides. The number of retrogradely labeled cells was determined in 25 μm-thick serial cryostat sections. To avoid double counting of neurons, present in two consecutive sections, the retrogradely labeled neurons were mapped and their locations were compared with those of labeled neurons in the previous section.

### Immuno- and lectin histochemistry

Nonspecific binding sites were blocked with 3% bovine serum albumin (BSA, Sigma-Aldrich, St. Louis, USA). Primary antibodies and lectin were used as follows: rat anti-M2 (mouse-specific astrocyte marker, DSHB, Toronto, Canada, 1:400), rat anti-M6 (mouse-specific neuron marker, DSHB, Toronto, Canada, 1:400), rabbit anti-VAChT (139 103, Synaptic Systems, Coventry, United Kingdom, 1:500), goat anti-Iba1 (ab5076, Abcam, Cambridge, United Kingdom, 1:500), rabbit anti-CD68 (MAB101141-100, BioTechne, Minneapolis, USA, 1:200) and biotinylated Griffonia Simplicifolia isolectin B4 (GSA-B4, B1205, Vector Laboratories, Newark, USA, 1:200) The immune reaction was completed by using donkey anti-rat Alexa Fluor 594 (A21209, Thermo Fisher Scientific, Waltham, USA, 1:600), donkey anti-rabbit Alexa Fluor 488 (A21206, Thermo Fisher Scientific, Waltham, USA, 1:600), donkey anti-goat Alexa Fluor 488 (A11055, Thermo Fisher Scientific, Waltham, USA, 1:600) and streptavidin Alexa Fluor 546 (S11225, Thermo Fisher Scientific, Waltham, USA, 1:600). Negative controls for the secondary antibodies were performed by omitting the primary antibodies. The sections were covered by using Vectashield mounting medium (Vector Laboratories, Newark, USA). Sections of interest were photographed using an Olympus BX-41 epifluorescence microscope equipped with a DP-74 digital camera and CellSens software (V1.18; Olympus, Tokyo, Japan).

### Quantification of microglia/macrophage densities

To assess the density of GSA-B4, Iba1 and CD68 reactivities in treated and control animals, the ventral horns of spinal cord were analyzed for each marker. Analysis was performed according to our earlier study [13, 14]. Five cross Sect. (250 μm apart from each other) were analyzed 3 days (GSA-B4: in AVR and AVR + 10^6^ NE-TR-4C animals) and 7 days (Iba-1 and CD68: in AVR and AVR + 100 mg Fucoidan, AVR + 100 mg Fucoidan and AVR + 10^6^ NE-TR-4C animals) after injury on the L4 spinal segment. Microphotographs were taken using an an Olympus BX-41 epifluorescence microscope equipped with a DP-74 digital camera and CellSens software (V1.18) and the affected ventral horn of spinal cord section area was analyzed by using the ImageJ software (NIH). The background intensity of unstained samples was individually subtracted from the intensity of treated sections. Iba-1, CD68 and GSA-B4 positive areas of the injured spinal cords were then divided by the size of examined area and multiplied by 100.

### Proteome profiler cytokine array

The spinal cords were collected 3 and 7 days after the injury and grafting. The rat spinal cord fractions were homogenized in PBS with protease inhibitor (Sigma-Aldrich, Burlington, United States). After homogenization Triton X-100 (Sigma-Aldrich, Burlington, United States) was added to a final concentration of 1%. The samples were frozen to -75 ºC, thawed and centrifuged at 10,000 x g for 5 min. The supernatant was collected and the total protein concentration was determined by using the Pierce BCA Protein Assay Kit (Thermo Fisher Scientific, Waltham, United States). The cytokine and chemokine contents of the samples (spinal cord and serum) were determined using the Proteome Profiler Rat Cytokine Array Kit, Panel A (R&D Systems, Minneapolis, USA). For the parallel determination of the relative levels of selected rat cytokines and chemokines, we used 390 µg of total protein of spinal cord homogenates on each membrane. The assay was performed following the manufacturer’s instructions. The signals from the bound cytokines present in the spinal cord were detected using the LI-COR Odyssey Imaging System (Lincoln, USA) and analyzed with the Image Studio Software (Lincoln, USA).

### RNA isolation and real-time polymerase chain reaction (qPCR)

In total 12 animals were used for qPCR evaluations. To study the gene expression changes of inflammatory components and mediators, the rats were allowed to survive to 7 days after ventral root avulsion injury. To assess the impact of fucoidan treatment on gene expression, animals from 3 groups were used (AVR, AVR + 50 mg Fucoidan, AVR + 100 mg Fucoidan).

After the appropriate postoperative time, the animals underwent transcardial perfusion with physiological saline, and the L4 spinal cord segment was removed and dissected. TRIzol reagent (15596026, Thermo Fisher Scientific, Waltham, USA) was used to homogenize the tissue samples, followed by isolation of total RNA using the Direct-zol RNA Miniprep Plus Kit (R2050, Zymo Research, Irvine, USA). Maxima first strand cDNA Synthesis Kit (K1672, Thermo Fisher Scientific, Waltham, USA) was used to transcribe RNS into cDNA. The amplification process was performed using iTaq™ Universal SYBR^®^ Green Supermix (K0381, Thermo Fisher Scientific, Waltham, USA) on a Bio-Rad CFX96 Real-Time PCR instrument (RRID: SCR_018064; Bio-Rad, Hercules, USA), adhering to the prescribed conditions: 40 × (95 °C/15 s, 60 °C/30 s, and 72 °C/30 s).

The primer sequences for each gene were as stated below:

IL1B Fw: TGGCAACTGTCCCTGAACTC, Rev: AAGGGCTTGGAAGCAATCCTT;

IL6 Fw: TCCGGAGAGGAGACTTCACA, Rev: GAATTGCCATTGCACAACTCTT;

TNFA Fw: GATCGGTCCCAACAAGGAGG, Rev: CTTGGTGGTTTGCTACGACG;

CCL3 Fw: GCTTCTCCTATGGACGGCA, Rev: CTCTTGGTCAGGAAAATGACACC;

NLRP3 Fw: TAGCTTCTGCCGAGGTCTCT Rev: GCAGCTGACCAACCAGAGTT;

NLRC4 Fw: AAGATGCTAAAAGCCTAGCGGA Rev: ATGTAGTCCATCCCCTCCCC.

### Biodistribution of NE-4C cells

The intravenously administered stem cells are capable of infiltrating various organs, adhering, and surviving for a certain period, making the investigation of this process fundamental. Carboxyfluorescein diacetate succinimidyl ester (CFSE, originally from Molecular Probes) was obtained from Thermo Fisher Scientific (Waltham, United States). The cells were labelled according to the instructions provided by the manufacturer. Briefly, CFDA-SA (10 µM) was added to the cells suspended in serum free medium at 10 million cells/ml, then the suspension was incubated for 10 min, at 37 °C. The reaction was stopped by adding cold medium and the cells were washed three times.

After the CFSe labelling, NE-TR-4C cells (1 × 10^6^) were intravenously administered for short term experiments. The spinal cord, lungs, liver and spleen were collected 1, 3 or 7 days after cell infusion and weighed. The tissues were postfixed for 1 day and then cryoprotected in 30% sucrose (in PBS). Serial 25 μm thick cryostat sections were cut from the L4 spinal segment. The spleen and the lungs were divided into 4 equal parts and weighed. Four equal parts were cut out from the liver and weighed. Serial 25 μm thick cryostat sections were made from each organ. Sections were stained with DAPI and the number of CFSE+/TR + stem cells were mapped and counted. Images were taken with an Olympus FluoView^®^ FV10i compact confocal microscope (Olympus, Tokyo, Japan). Data are expressed as number of cells/gram tissue.

### Analysis of locomotion pattern

During the survival time video-based kinematic analysis was carried out [15]. The hair of the rats was shaved off from the hind limbs and the skin was marked with a black pen above the major joints. We used a plexiglass runway equipped with a mirror system in order to record the position of the hind limb from both lateral and rear-view aspects. Two high resolution and high-speed cameras (GoPro, The Imaging Source, Bremen, Germany) were used to during 3 to 4 step cycles. By comparing specific single video frames, we measured six different parameters to get detailed information on the gate improvement. The animals were trained prior to the measurements to walk from one end of the runway to the other reaching a shelter and were tested every week postoperatively.

### Statistical analysis

The paired T-test or one-way ANOVA test followed by Tukey’s all pairwise multiple comparison procedure were used to compare the data. The level of significance was set at *p* < 0.05, and all error bars represent the standard error of mean (SEM).

## Results

### Dose dependent effect of systemic NE-TR-4C cell treatment on the survival and regeneration of injured motoneurons

**Fig. 1:**
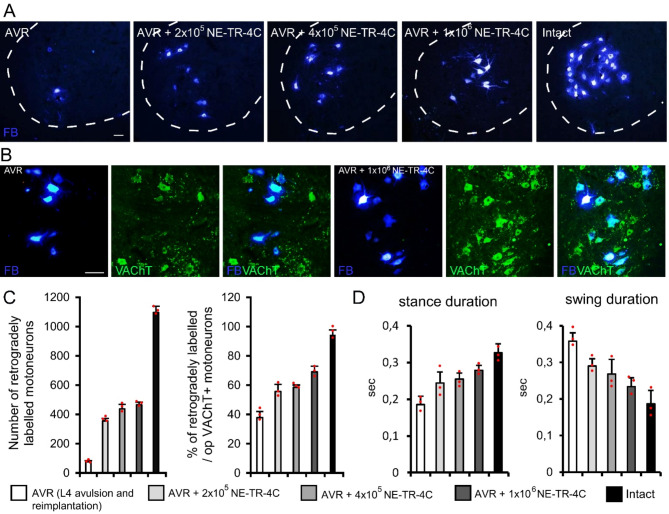
The survival and regeneration of injured motoneurons exhibit a dose-dependent effect following systemic NE-TR-4C cell treatment. **(A)** The avulsion and reimplantation of the L4 ventral root (AVR) led to a notable reduction in the numbers of surviving and reinnervating motoneurons. **(B)** Representative images showing the reinnervating motoneurons colocalized with VAChT. **(C)** Intravenous administration of NE-TR-4C cells resulted in significantly increased numbers of reinnervating motoneurons in the L4 spinal segment compared to the AVR group. Nearly 60% of the surviving motor neurons were able to reinnervate the reimplanted ventral root in the stem cell-treated groups, whereas in the control group (AVR), this ratio barely reached 40%. **(D)** Functional analysis revealed that animals treated with intravenous stem cells exhibited an improved movement pattern comparable to that of the intact hind limb. The duration of stance and swing phases in AVR animals significantly differed from those in the groups treated with stem cells. Dashed lines indicate the borderline between the white and grey matter. Data are expressed as mean ± SEM. Asterisk indicates significant difference between AVR vs. AVR + 2 × 10^5^ NE-TR-4C and AVR + 4 × 10^5^ NE-TR-4C and AVR + 10^6^ NE-TR-4C. * *p* < 0.05; (*n* = 3 animals/group) Scale bar in A and B: 50 μm. Abbreviations: FB: Fast Blue, VAChT: vesicular acetylcholine transporter

The retrogradely labelled motoneurons (FB/VAChT double labelled cells) were localized in the ventral horn of the L4 spinal segment (Fig. 1A-B). In the intact L4 motoneuron pool, the average number of the retrogradely labelled motoneurones was 1161 ± 56 (SEM; Fig. 1C). Avulsion and reimplantation of the L4 ventral root resulted in a significant decrease in surviving and reinnervating motoneurones numbers (Fig. 1A, C). The average number of retrogradely labelled motoneurones was only 57 ± 11 in the AVR group (SEM; Fig. 1A, C).

In the next series of experiment, the dose dependent effect of intravenously grafted NE-TR-4C stem cells was studied following L4 ventral root avulsion and reimplantation. Various doses of NE-TR-4C cells (2 × 10^5^, 4 × 10^5^ or 10^6^) were injected into the blood stream immediately after the injury. Intravenous administration of NE-TR-4C cells resulted in significantly higher numbers of reinnervating motoneurons (AVR + 2 × 10^5^ NE-TR-4C group: 355 ± 24; AVR + 4 × 10^5^ NE-TR-4C group: 423 ± 46; AVR + 1 × 10^6^ NE-TR-4C group: 444 ± 35, SEM) in the L4 spinal segment compared with the AVR group (Fig. 1A, C). Although the intravenous grafting of 10^6^ NE-TR-4C cells produced the highest number of reinnervating motoneurons, there was no significant difference in the number of FB-positive reinnervating motoneurons between AVR + 4 × 10^5^ NE-TR-4C and AVR + 1 × 10^6^ NE-TR-4C animals (Fig. 1C). These results show that almost 36% of the total population of L4 motoneurons was able to survive and sent their axons into the reimplanted L4 ventral root after systemic NE-TR-4C cell treatment. To assess the proportion of surviving motoneurons that extended their axons into the reimplanted L4 ventral root, we compared the number of retrogradely labeled, VAChT-positive motoneurons with the total number of surviving VAChT-positive motoneurons in the injured L4 segment on the operated side. In the AVR rats, only 37 ± 4% of the surviving VAChT-positive motoneurons were able to reinnervate the L4 ventral root, as indicated by the presence of FB-positive motoneurons. In contrast, in the stem cell-treated animals, the reinnervation percentage was significantly higher (56 ± 5% in the AVR + 2 × 10^5^ NE-TR-4C group, 58 ± 2% in the AVR + 4 × 10^5^ NE-TR-4C group, 69 ± 4% AVR + 1 × 10^6^ NE-TR-4C group). This suggests that in the stem cell-treated groups, well over 50% of the surviving motoneurons were able to send their axons into the reimplanted L4 ventral root and peripheral nerve. Morphological observations were confirmed by quantitative gait analysis. Animals that received intravenous stem cell treatment developed an improved movement pattern similar to that of the intact hind limb (Fig. 1E). Duration of stance and swing phases in AVR animals differed significantly from those of the stem cell-treated groups (Fig. 1D).

### NE-TR-4C stem cell derivatives in the L4 injured segment 3 months after the injury

**Fig. 2:**
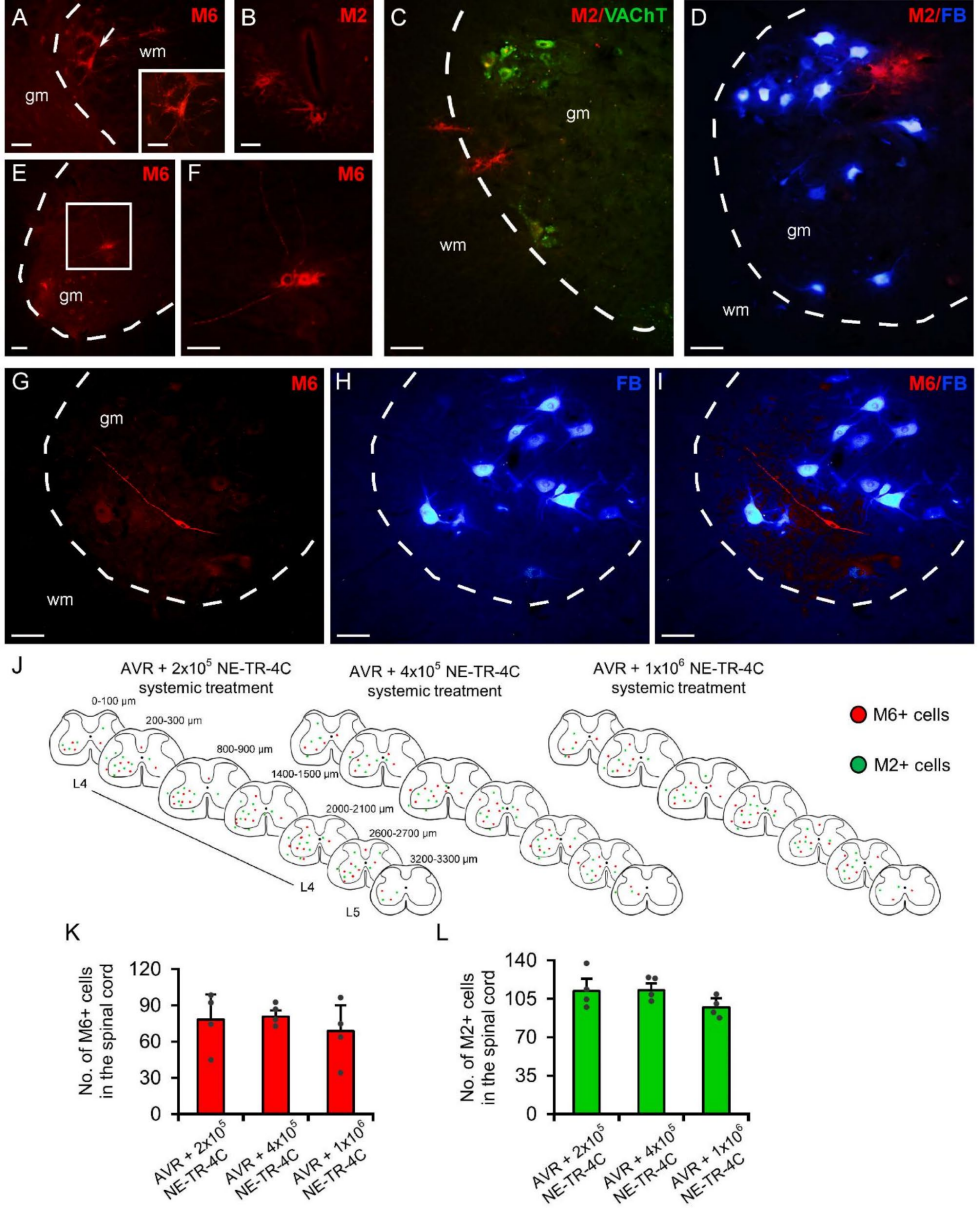
Distribution of NE-TR-4C stem cell derivatives in the spinal cord 12 weeks after the injury. **(A-C)** Stem cell derivatives (M6 + neurons and M2 + astrocytes) were localized at the border of the white and grey matter of the spinal cord and were observed close to the central canal. **(D)** Stem cell-derived astrocytes appeared in the environment of retrogradely labelled host motoneurons (FB+). (**E-F**) Rarely stem cell-derived M6 + neurons with long processes were found in the affected ventral horn. (**G-I**) Stem cell-derived neurons (M6+) were observed surrounding retrogradely labeled host motoneurons. No colocalized cells were found. **(J)** Rostro-caudal distribution of M2-positive stem cell-derived astrocytes and neurons in various experimental groups. Low numbers of stem cell-derivatives were dispersed consistently in the injured side of the L4 segment. **(K-L)** Diagrams show the number of M6/+ and M2 + cells in the injured spinal segment. Dashed lines indicate the borderline between the white and grey matter. Arrows show stem cell-derivatives. wm = white matter, gm = grey matter, Data are expressed as mean ± SEM. (*n* = 4 animals/group) Scale bar in A: 50 and 25 μm, in B: 25 μm, in C-D: 50 μm, in E-F: 50 μm, in G-I: 30 μm

Twelve weeks after the surgery and intravenous administration of stem cell, the NE-TR-4C cell-derivatives were mapped by immunohistochemistry. Although various number of NE-TR-4C cells was applied, there was no remarkable difference between morphological appearance or distribution of stem cell derivatives observed in each group. Analysis of cross sections through the L4 segment revealed that the vast majority of the stem cell-derived neurons (M6+) and astrocytes (M2+) were found in the affected spinal segment (Fig. 2A-I). Few stem cell-derived neurons and astrocytes appeared at the gray and white matter interface close to the dorsal horn or the central canal (Fig. 2A-C). The majority of these cells settled down in the injured ventral horn and showed various morphological appearance (Fig. 2A-I). Stem cell-derived neurons were often characterized by numerous processes, but some of them showed a fusiform morphology (Fig. 2E-G) and settled close to the motoneurons (not shown), sometimes forming contact with the proximal dendrites of retrogradely labelled cells (Fig. 2G-I). Stem cell-derived neurons had relatively small cell bodies, and occasionally, cells with particularly long processes appeared (Fig. 2E-I). The M6 + and M2 + cells were dispersed rostro-caudally within the injured L4 spinal segment but appeared in small number in the L3 and L5 segments, too (Fig. 2J). Although various numbers of NE-TR-4C cells were applied intravenously immediately after the injury, no significant difference was found in the number of stem cell derivatives among the stem cell-treated groups 3 months after the injury (Fig. 2K-L).

### Systemic distribution of NE-TR-4C cells after intravenous infusion

**Fig. 3:**
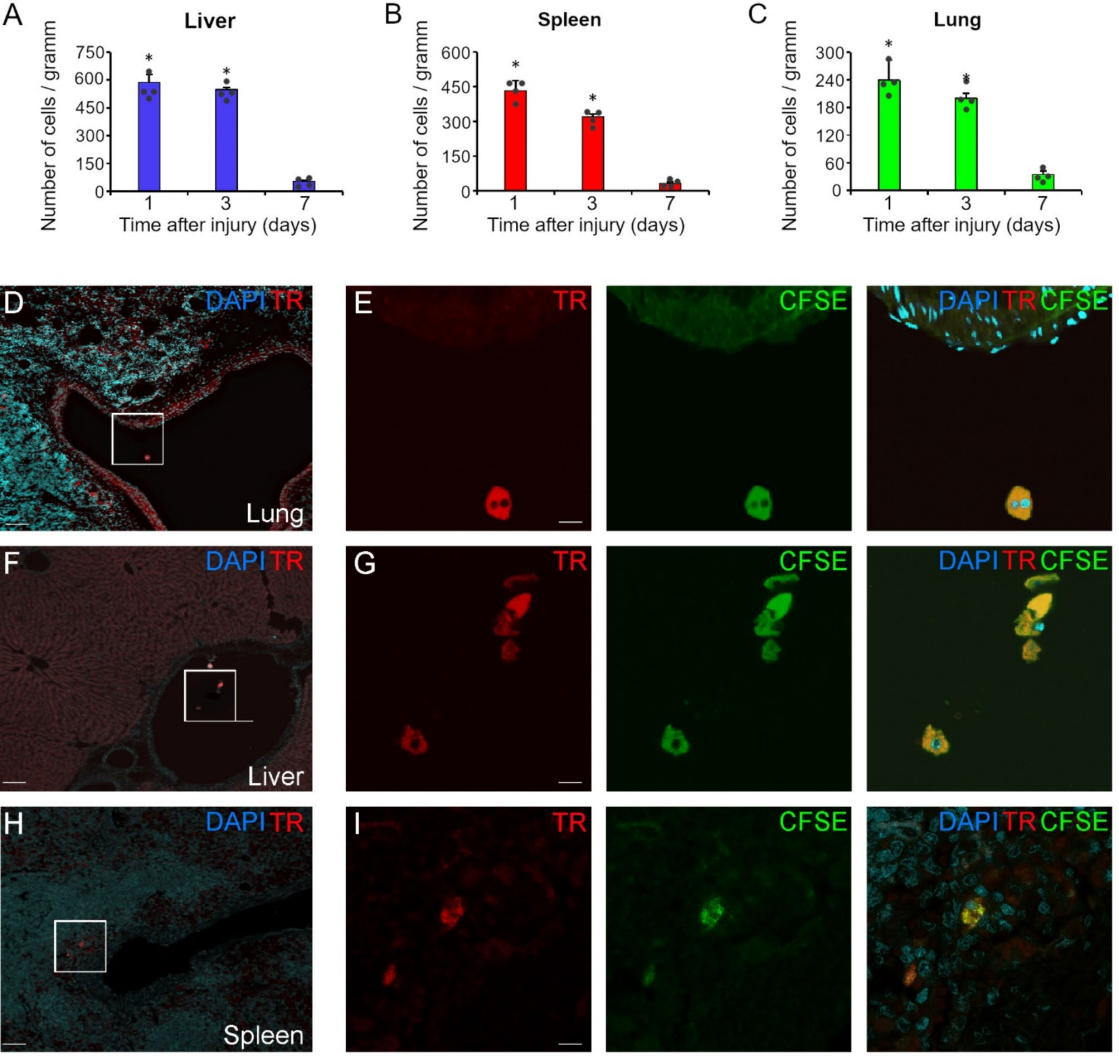
Temporal biodistribution of NE-TR-4C stem cells. **(A-C)** Systemic delivery of NE-TR-4C stem cells reveals rapid cell clearance. A dramatic drop in the number of Tomato Red/CFSE-labeled cells can be observed in all organs investigated. **(D-I)** Representative images show NE-TR-4C cells co-localized with CFSE in the lung, liver and spleen 1 day after the cell infusion. Framed areas in in E, G and I are shown in the panels of D, F and H, respectively. The number of NE-TR-4C cells per gram tissue is expressed as mean ± SEM. Asterisk indicates significant difference between d1 vs. d7 and d3 vs. d7. in A, B, C. (*n* = 4 animals/group) Scale bar in D, F and H: 200 μm and in E, G, and I: 10 μm. Abbreviation: CFSE: carboxyfluorescein diacetate succinimidyl

Since the best morphological and functional results were obtained after the systemic administration of 10^6^ NE-TR-4C stem cells, we continued to work with this set up. To determine the fate of the stem cells after intravenous administration, the spleen, lungs, and liver were removed 1, 3, and 7 days after intravenous grafting. By the end of the 1st day, limited numbers of NE-TR-4C cells were found in the liver (587 ± 44), in the spleen (432 ± 34) and in the lung (239 ± 22; Fig. 3A-C). On day 3 moderate decrease was observed in number of NE-TR-4C cells in all examined organs (liver: 549 ± 11; spleen: 321 ± 20; lung: 200 ± 25, Fig. 3A-C). On day 7 a marked decrease was seen in the number of stem cells in any of the samples examined (liver: 53 ± 7; spleen: 32 ± 4; lung: 35 ± 15). Interestingly, the NE-TR-4C cells were also found within blood vessels and failed to enter the parenchyma of the organs (Fig. 3D-G). Stem cells co-localizing TR and CFSE were most likely found near the large vessels in the sinusoidal system of the spleen (Fig. 3H-I). Furthermore, it is important to note that no NE-TR-4C cells were found in the spinal cord at any examined time point (data not shown). These results suggested that NE-TR-4C cells may have an impact on the regenerative effect of the motoneurons through an unknown humoral effect.

### Cytokine changes and microglia/macrophage reaction after intravenous administration of NE-TR-4C cells

**Fig. 4:**
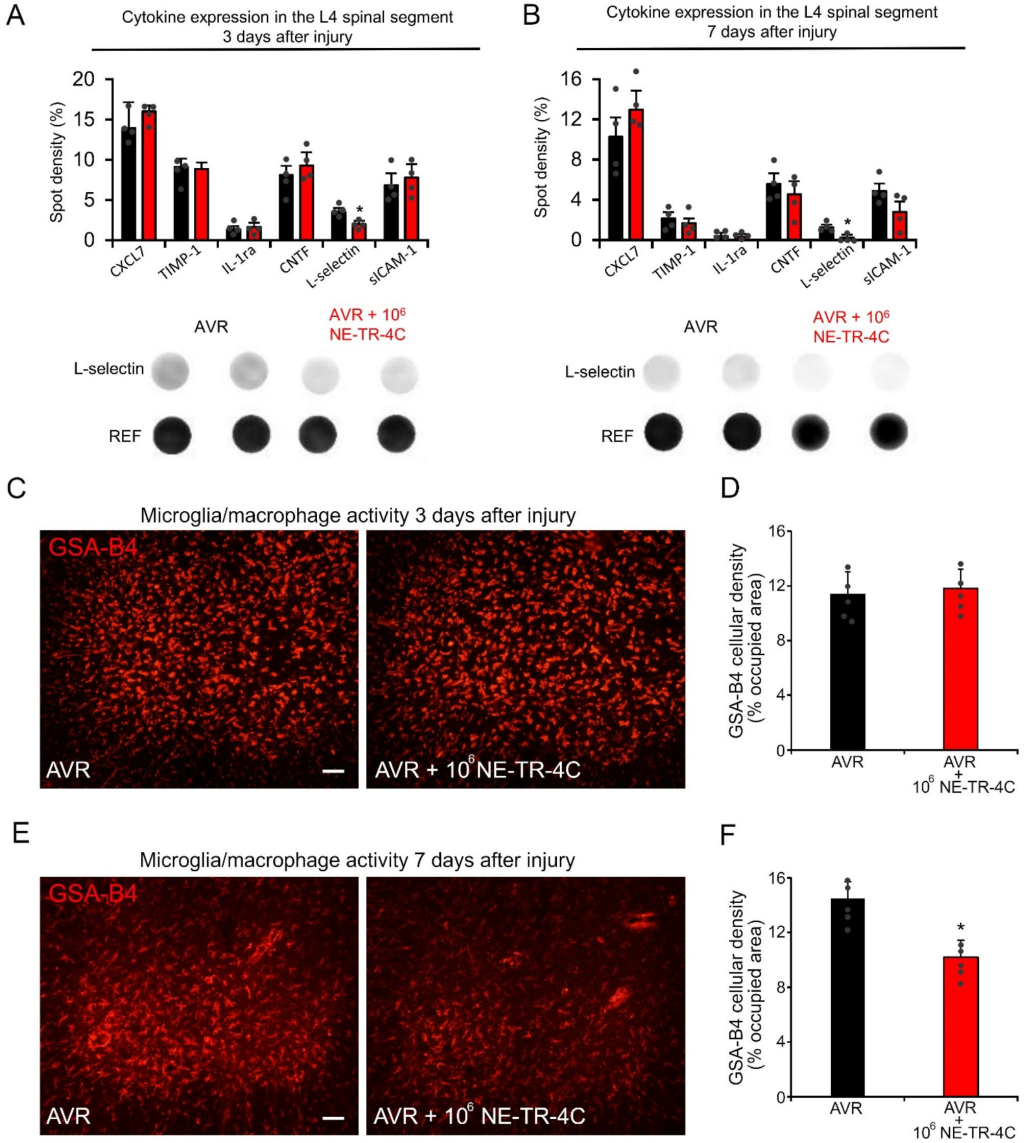
Rat chemokine/cytokine and microglia/macrophages changes after intravenous NE-TR-4C stem cell infusion in L4 spinal segment. **(A-B)** Proteome Profiler array provides data on the relative levels of 29 chemokine/cytokines. Significant differences between the experimental groups (AVR and AVR + 10^6^ NE-TR-4C) are shown for L-selectin. **(C)** Representative images display GSA-B4-positive cells in the L4 segment from AVR and AVR + 10^6^ NE-TR-4C animals 3 days after the injury. **(D)** Quantification of GSA-B4 cellular density reveals no significant difference between the experimental groups 3 days after the injury. **(E)** Representative images of GSA-B4-positive cells are shown 7 days after the injury. **(F)** The quantified density of GSA-B4 was significantly lower in the AVR + 10^6^ NE-TR-4C group compared with AVR group. Dashed lines indicate the borderline between the white and grey matter. Data are expressed as mean ± SEM. Asterisks indicate significant difference between AVR vs. AVR + 10^6^ NE-TR-4C group. * = *p* < 0.05; (*n* = 4 animals/group in Fig. A and B; *n* = 5 animals/group in Fig. D and F) Scale bar in C and E: 50 μm. Abbreviations: CXCL7 (Chemokine C-X-C Motif Ligand 7); TIMP-1 (tissue inhibitor matrix metalloproteinase 1); CNTF (ciliary neurotrophic factor); IL-1ra (interleukin-1 receptor antagonist protein); sICAM-1 (soluble intercellular adhesion molecule-1)

To investigate whether intravenously applied stem cells affect the microenvironment of the damaged motoneurons, cytokine changes within the spinal cord were evaluated with the R&D ELISA Proteome Profiler array 3 and 7 days after avulsion and reimplantation. A significant decrease within L-selectin level was detected in AVR + 10^6^ NE-TR-4C group on days 3 and 7 compared with the AVR group (Fig. 4A and B).

In our earlier study, we have shown that intravenous administration of NE-4C cells induced a decrease of the microglia/macrophage activity following spinal cord contusion injury [11]. Therefore, we next investigated the microglia/macrophage reaction in the ventral horn of AVR and AVR + 10^6^ NE-TR-4C animals 3 and 7 days after the injury. GSA-IB4 is an overall marker for microglia/macrophage cells, in comparison to Iba1 and CD68 [16]. On day 3 no significant difference was found in the cellular density of GSA-B4 isolectin between the AVR and AVR + 10^6^ NE-TR-4C groups (Fig. 4C and D). In contrast, microglia/macrophage activities decreased significantly by day 7 throughout the L4 segment in AVR + 10^6^ NE-TR-4C animals compared with the AVR animals (Fig. 4E and F).

### Fucoidan and intravenous NE-TR-4C stem cell treatment reduces microglia and macrophage activity in the injured L4 spinal segment

Based on the above results the question was raised, whether blocking of selectin expression and thus likely reducing the microglia/macrophage reaction in the injured spinal cord could induce the same neuroprotection and regeneration following ventral root avulsion and reimplantation as intravenous administration of NE-TR-4C stem cells. To investigate our hypothesis, we used fucoidan, a promising compound proven to block selectins, in two doses (50 and 100 mg) i.p. after injury.

**Fig. 5:**
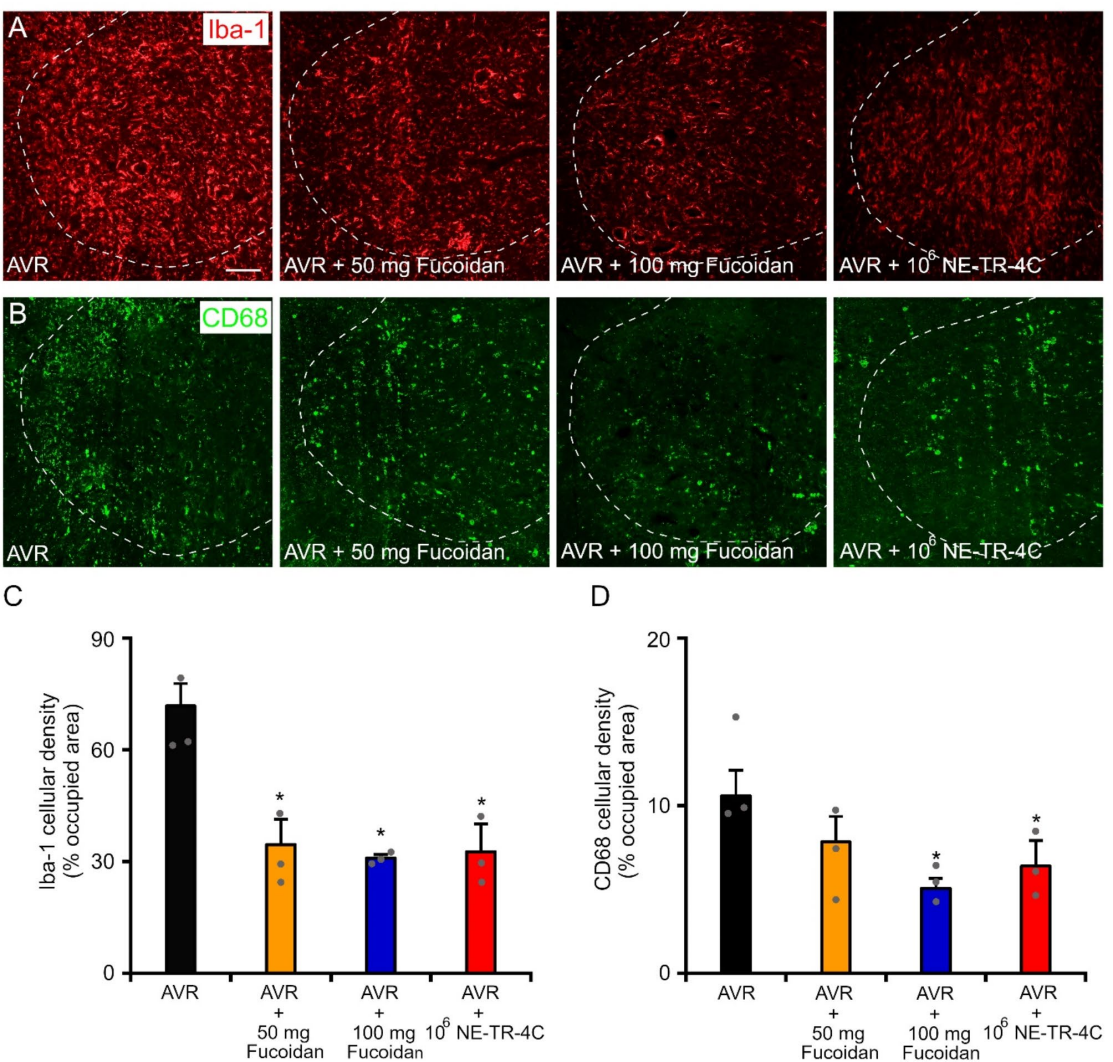
Fucoidan treatment decreases Iba-1 and CD68 densities. **(A**,** B)** Representative images of cross sections (affected side of L4 spinal segment) show Iba-1 (A) and CD68 reactivity (B) 7 days after injury in various experimental groups (AVR, AVR + 50 mg Fucoidan, AVR + 100 mg Fucoidan and AVR + 10^6^ NE-TR-4C). **(C)** Quantification of microglia (Iba-1) densities in the cross sections of the spinal cord revealed significantly decreased level of Iba-1 at 7days after the injury in the fucoidan and NE-TR-4C cell-treated groups compared with the AVR group. **(D)** Quantification of CD68 density in the cross section of L4 spinal segment displays significantly decreased level of CD68 only in AVR + 100 mg Fucoidan and AVR + 10^6^ NE-TR-4C group compared with the AVR group. Dashed lines indicate the borderline between the white and grey matter. Data are expressed as mean ± SEM. Asterisk indicates significant difference between AVR vs. AVR + 50 mg Fucoidan and AVR + 100 mg Fucoidan and AVR + 10^6^ NE-TR-4C in C. Asterisk indicates significant difference between AVR vs. AVR + 100 mg Fucoidan and AVR + 10^6^ NE-TR-4C in D. * *p* < 0.05; (*n* = 3 animals/group) Scale bar in A: 50 μm

The effect of fucoidan treatment starting immediately after the operation was studied next. Strong microglial reaction (Iba-1) was present in the affected L4 spinal segment in the control (AVR) group, whereas fucoidan or stem cell treatment in each treated group (AVR + 50 mg fucoidan, AVR + 100 mg Fucoidan and AVR + 10^6^ NE-TR-4C group) significantly diminished the microglial reaction 7 days after the injury (Fig. 5A and C). Similar to microglia reaction (Iba1+), the CD68-positive macrophage activity was also markedly reduced by 50 mg Fucoidan treatment, but a significant decrease of CD68 density was only observed in the case of 100 mg fucoidan or NE-TR-4C stem cell administration (Fig. 5B and D). Based on the results above, the fucoidan-treated groups (AVR + 50 mg fucoidan, AVR + 100 mg fucoidan) can be regarded as a positive control for the stem cell-treated group (AVR + 10⁶ NE-TR-4C).

### Fucoidan treatment modulates gene expression of inflammatory components and mediators

**Fig. 6:**
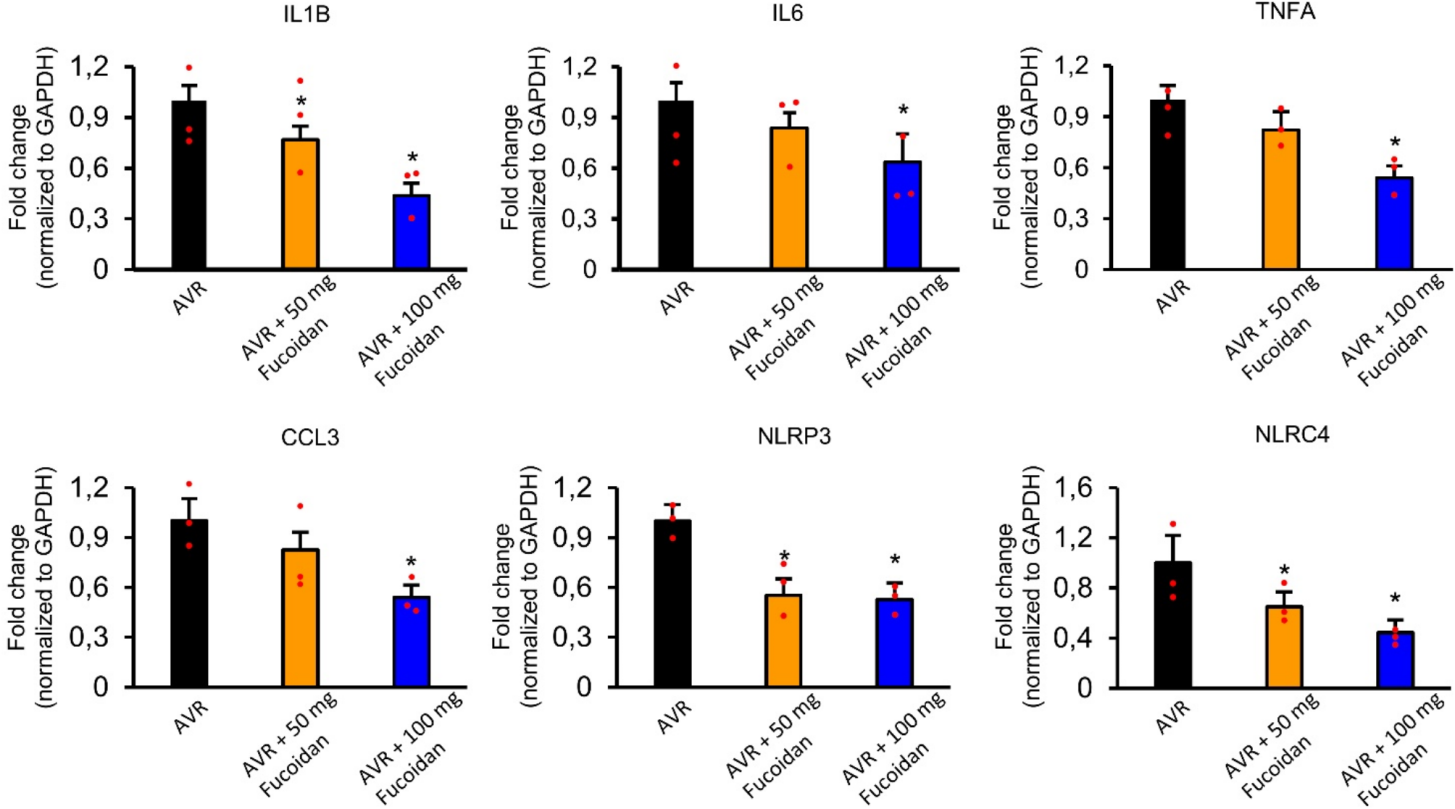
Gene expression changes of inflammatory components in the L4 spinal segment following fucoidan treatment. Changes in the mRNA levels of IL1B, IL6, TNFA, CCL3, NLRP3 and NLRC4 7 days after ventral root avulsion injury, as assessed by qPCR are shown. Fucoidan treatment in the AVR + 100 mg Fucoidan group markedly decreased the mRNA levels of various proinflammatory molecules. Data are expressed as mean ± SEM. Asterisk indicates significant difference between AVR vs. AVR + 50 mg Fucoidan and AVR + 100 mg Fucoidan. * *p* < 0.05; (*n* = 3 animals/group) Abbreviations: IL1B (interleukin-1b); IL6 (interleukin-6), TNFA (tumor necrosis factor alpha); CCL3 (C-C Motif Chemokine Ligand 3), NLRP3 (NLR family pyrin domain containing 3); NLRC4 (NLR family CARD domain containing 4)

The gene expression changes of inflammatory factors were analyzed 7 days after the injury through the use of qPCR. The lower dose of fucoidan treatment (AVR + 50 mg Fucoidan group) caused remarkable, but not always significant decrease of mRNA levels of the various proinflammatory molecules compared with the AVR group (Fig. 6). Higher dose of fucoidan (AVR + 100 mg fucoidan group) administration induced significant downregulation of gene expression for proinflammatory cytokines/chemokine (IL1B, IL6, TNFA and CCL3) and reduced the pattern recognition receptor expression (NLRP3, NLRC4) compared with control animals (AVR group) (Fig. 6).

### Fucoidan treatment rescues injured motoneurons and induces morphological and functional recovery

**Fig. 7:**
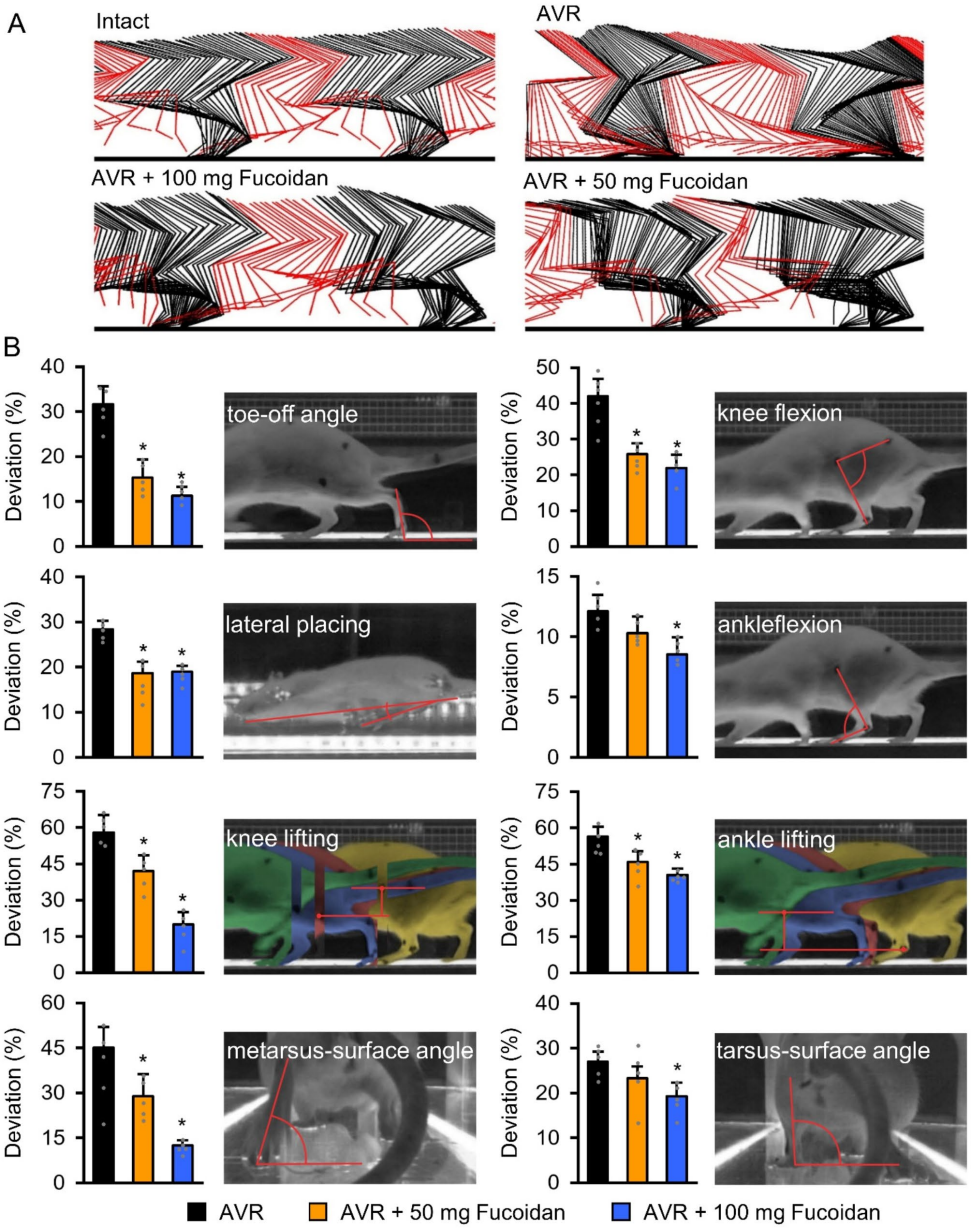
Kinematic analysis of various experimental groups. **(A)** Representative kinematic patterns during locomotion 11 weeks after injury are shown as a stick diagram decomposition of hindlimb motion. **(B)** Note the significantly improved parameters of the fucoidan-treated animals (AVR + 50 mg Fucoidan and AVR + 100 mg Fucoidan group) compared with the AVR group. Data are expressed as mean ± SEM. *p < 0.05 and indicates significant difference between AVR vs. AVR + 50 mg Fucoidan and AVR + 100 mg Fucoidan group, respectively. #*p* < 0.05 and shows significant difference between AVR vs. AVR + 100 mg Fucoidan group. (*n* = 5 in each group)

Fucoidan treatment started immediately after the injury and lasted for 2 weeks. All rats exhibited evaluable locomotion pattern at the ankle the knee joints 11 weeks after the injury (Fig. 7A). Detailed kinematic analysis of fucoidan-treated (AVR + 50 mg vs. 100 mg Fucoidan) groups and control rats (AVR groups) was performed to provide quantitative information about knee flexion and lifting, ankle flexion and lifting, range of knee flexion and ankle flexion, metatarsus surface angle and tarsus surface angle. Our analysis revealed that fucoidan-treated animals displayed significant improvement in all examined parameters except ankle flexion and tarsus surface angle in AVR + 50 mg Fucoidan group compared with control animals (AVR group) which displayed only minimal recovery after avulsion injury (Fig. 7B).

**Fig. 8:**
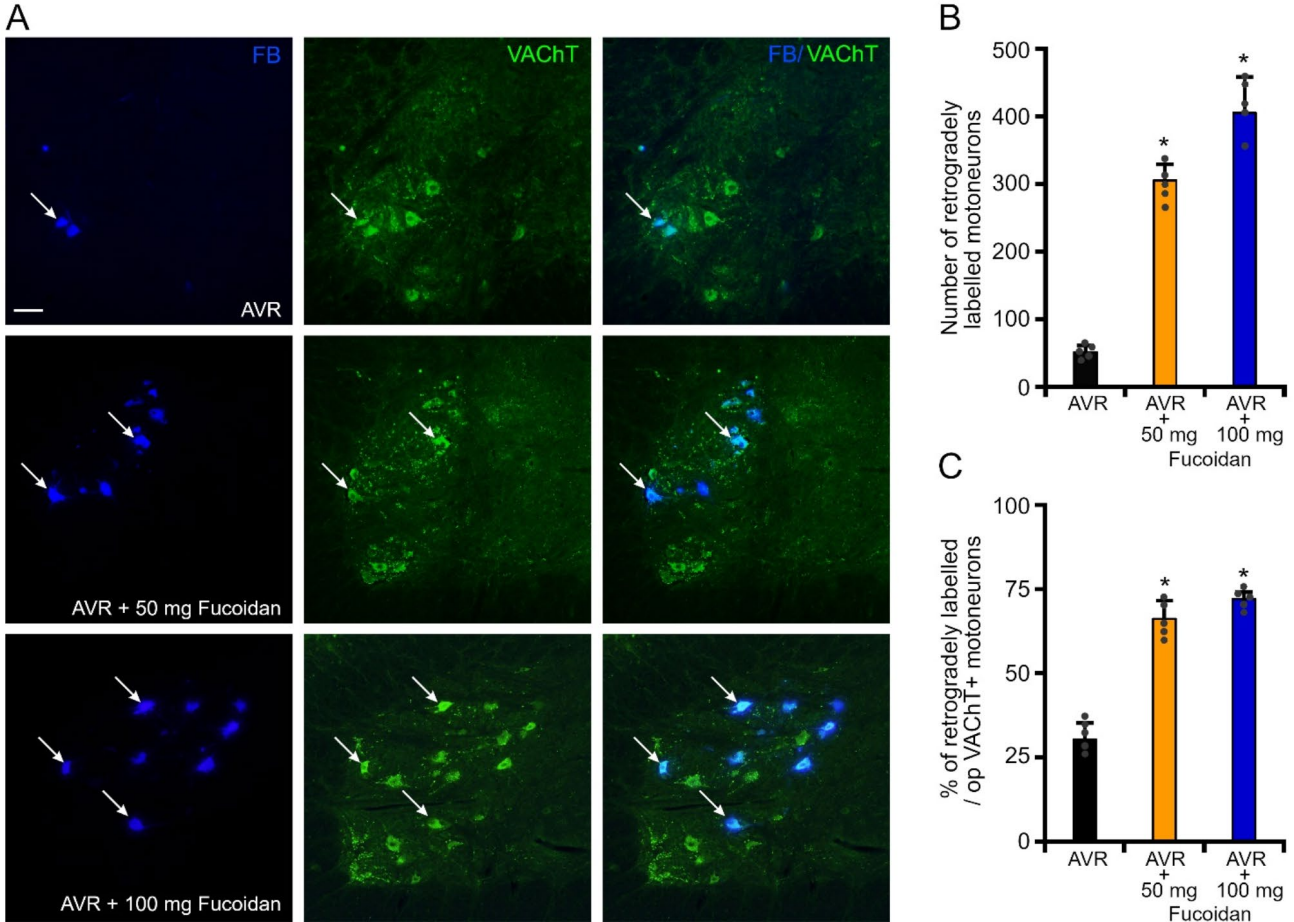
Retrogradely labelled (FB+) and surviving motoneurons (VAChT+) in the various experimental groups (AVR, AVR + 50 mg Fucoidan and AVR + 100 mg Fucoidan) 12 weeks after injury. **(A)** Representative images show FB + and VAChT + motoneurons in all three experimental groups. **(B)** Significantly higher numbers of reinnervating L4 motoneurons (FB+) were able to reinnervate the reimplanted ventral root in the fucoidan-treated groups (50 mg vs. 100 mg fucoidan treatment) compared with control animals (AVR). **(C)** Significantly higher number of surviving motoneurons were found in the fucoidan-treated animals (AVR + 50 mg Fucoidan and AVR + 100 mg Fucoidan) compared with controls (AVR). Arrows show FB+/VAChT + co-labelled motoneurons. Data are expressed as mean ± SEM. Asterisk indicates significant difference between AVR vs. AVR + 50 mg Fucoidan and AVR vs. AVR + 100 mg Fucoidan groups. * = *p* < 0.05. (*n* = 5 animals/group) Scale bar in A: 50 μm

After kinematic gait analysis the L4 motoneuron pool was retrogradely labeled with FB and the number of reinnervating motoneurons was determined. In animals, whose avulsed L4 ventral root was reimplanted into the injured cord without treatment (AVR group), 50 ± 10 retrogradely-labeled motoneurons were found (Fig. 8A-B). In the experiments with 50 mg or 100 mg i.p. fucoidan treatment, significantly higher numbers of retrogradely labelled motoneurons (305 ± 10 in AVR + 50 mg Fucoidan, 405 ± 72 in AVR ± 100 mg Fucoidan) were counted compared with AVR animals (Fig. 8A-B). Although the number of retrogradely labelled motoneurons appeared to be somewhat higher in AVR ± 100 mg Fucoidan animals, there was no significant difference in the reinnervating motoneuron numbers between AVR + 50 mg Fucoidan and AVR ± 100 mg Fucoidan animals.

In order to determine the proportion of surviving motoneurons that sent their axons into the reimplanted L4 ventral root, the number of retrogradely labelled VAChT-positive motoneurons was compared with the number of surviving VAChT-positive motoneurons in the injured L4 segment on the operated side. In the AVR rats, the percentage of reinnervating FB-positive motoneurons within the surviving VAChT-positive motor pool was only 30 ± 5%, whereas in the fucoidan-treated animals this ratio was significantly higher (66 ± 5% in AVR + 50 mg Fucoidan group, 72 ± 2% in AVR + 100 mg Fucoidan group), indicating that more than two thirds of the surviving motoneurons in the fucoidan treated groups were able to send their axon in the L4 reimplanted ventral root and peripheral nerve (Fig. 8C).

## Discussion

In this study we have clearly shown that intravenous administration of NE-TR-4C cells or i.p. administration of fucoidan following ventral root avulsion and reimplantation induced a significant motoneuron survival and functional reinnervation of denervated hind limb muscles.

As the appropriate dose of transplanted cells is always an interesting question, we compared the effect of intravenously administered different numbers of NE-TR-4C cells (2 × 10^5^, 4 × 10^5^ or 1 × 10^6^) on motoneuron survival and functional reinnervation following ventral root avulsion and reimplantation. Recent studies have already proven, that high doses of grafted stem cells may lead to higher efficacy on cell survival and improved functional outcome [17, 18]. We hypothesized that, by intravenous administration of incremental numbers of NE-TR-4C stem cells, the trophic effect of the grafted cells can be substantially increased. Indeed, our long-term experiments have provided evidence that infusion of 1 × 10^6^ cells resulted in the greatest motoneuron survival and ventral root reinnervation without significant difference in the number of reinnervating motoneurons as compared with grafting of 2 × 10^5^ or 4 × 10^5^ NE-TR-4C cells. Furthermore, only less than 0.1% of the total number of grafted undifferentiated NE-TR-4C cells were deposited as stem cell derivatives within the L4 spinal segment 12 weeks after the injury and this was not affected by the different doses of grafted cells.

In our earlier study we have provided evidence that intraspinally grafted undifferentiated NE-4C cells produced a so-called lesion-induced secretome that induced robust neuroprotection and functional reinnervation following ventral root avulsion or spinal cord contusion injury [2, 3]. This effect was manifested via a paracrine mechanism. Similarly, systemic administration of NE-4C cells following ventral root avulsion injury was also able to induce a remarkable survival of injured motoneurons and reinnervation of reimplanted ventral root, but without the invasion of significant numbers of the grafted cells. The transplanted NE-TR-4C cells were rapidly cleared from the blood circulation and the absence of any significant aggregation of neuroectodermal stem cells in the parenchyma of various organs (liver, spleen, lung or spinal cord) suggests that their neuroprotective effect is not elicited by direct action or paracrine signalling of the cells but more likely by systemic cell signalling. This hypothesis was confirmed by the proteome profiler results and microglia/macrophage analysis. In fact, the intravenous stem cell infusion induced a marked decrease of L-selectin in the injured spinal cord segment and a significantly decrease in the microglia/macrophage reaction, too.

L-selectin is a glycoprotein constitutively expressed by all leukocytes [19, 20]. It binds reversibly to its endothelial cell ligands and mediates leukocytes rolling on the endothelium during inflammation [21]. Inflammation promotes conformation changes of L-selectin leading to increased binding affinity to its ligands [22]. Due to the enhanced binding affinity leukocytes enter the parenchyma effectively after the injury. In the injured cords of stem cell-treated animals, the decreased L-selectin expression may have contributed to the decreased microglia/macrophage reaction. These results indicate that L-selectin plays a crucial role in the survival of injured motoneurons and the downregulated microglia/macrophage activity results in significant neuronal survival and axonal regeneration [1, 2, 11].

Considering the findings mentioned earlier, the significance of selectins appears to be further justified as they are reportedly expressed on leukocytes and endothelial cells following activation [23]. Recent evidence suggests that selectins regulate inflammation in various diseases [24]. To clarify the role of selectins in motoneuronal survival, fucoidan extracted from brown sea weeds was used in this study. Fucoidan acts as a ligand for either L- or P-selectin, thereby inhibiting leukocyte rolling [25]. Our study provided evidence that i.p. administration of fucoidan effectively decreased the density of CD68-positive macrophages in the affected L4 spinal segment compared with injured untreated animals. CD68 is highly expressed by monocytes and macrophages and can bind to selectins allowing homing of macrophages [26, 27]. Furthermore, Iba-1-positive microglia cell density was also significantly decreased indicating a strong correlation between microglia and macrophage activity.

Our results also showed that gene expression of the inflammasome component NLRP3 was markedly decreased by fucoidan treatment. These findings indicate the important role of NLPRP3 inflammasome component in the pathogenesis of motoneuron cell death and/or inflammation following ventral root avulsion injury. Although it is not clearly understood yet, which molecular pathway of inflammasome activation is affected by fucoidan treatment our results may suggest that decreased inflammasome activation most likely contributes to the survival of damaged motoneurons. This view is also supported by our previous study, where in the case of peripheral nerve injury inflammasome activation increased significantly in the affected motoneurons and their environment, but blocking of the inflammasome assembly significantly promoted functional reinnervation by the regenerating motoneurons [28].

## Conclusions

Taken together, our results clearly show that rendering the selectins defunct in a damaged spinal cord segment decreases the neuroinflammation in the affected spinal segment and induces significant motoneuron survival and functional reinnervation of the denervated hind limb muscles. Furthermore, inflammasome activation induced by avulsion injury could also be down regulated by fucoidan treatment.

## Data Availability

Not applicable.

## Abbreviations

AVR: avulsion and reimplantation of the L4 ventral root
CCL3: C-C Motif Chemokine Ligand 3
CFSE: carboxyfluorescein diacetate succinimidyl ester
CNTF: ciliary neurotrophic factor
CXCL7: Chemokine C-X-C Motif Ligand 7
FB: Fast Blue
gm: grey matter
IL1B: interleukin-1b
IL: 1ra-interleukin-1 receptor antagonist protein
IL6: interleukin-6
i.p.: intraperitoneal
L4: lumbar 4
NLRC4: NLR family CARD domain containing 4
NLRP3: NLR family pyrin domain containing 3
sICAM-1: soluble intercellular adhesion molecule-1
TIMP-1: tissue inhibitor matrix metalloproteinase 1
TNFA: tumor necrosis factor alpha
TR: Tomato Red
VAChT: vesicular acetylcholine transporter
wm: white matter

## References

[1] Pajer K, Nemes C, Berzsenyi S, Kovács KA, Pirity MK, Pajenda G, Nógrádi A, Dinnyés A. Grafted murine induced pluripotent stem cells prevent death of injured rat motoneurons otherwise destined to die. Exp Neurol. 2015;269:188–201. 25889458 10.1016/j.expneurol.2015.03.031

[2] Pajer K, Feichtinger GA, Márton G, Sabitzer S, Klein D, Redl H, Nógrádi A. Cytokine signaling by grafted neuroectodermal stem cells rescues motoneurons destined to die. Exp Neurol. 2014;261:180–9. 24907401 10.1016/j.expneurol.2014.05.026

[3] Pajenda G, Hercher D, Márton G, Pajer K, Feichtinger GA, Maléth J, Redl H, Nógrádi A. Spatiotemporally limited BDNF and GDNF overexpression rescues motoneurons destined to die and induces elongative axon growth. Exp Neurol. 2014;261:367–76. 24873730 10.1016/j.expneurol.2014.05.019

[4] Nógrádi A, Szabó A, Pintér S, Vrbová G. Delayed riluzole treatment is able to rescue injured rat spinal motoneurons. Neuroscience. 2007;144:431–8. 17084537 10.1016/j.neuroscience.2006.09.046

[5] Nógrádi A, Vrbova G. Reinnervation of denervated hindlimb muscles by axons of grafted motoneurons via the reimplanted L4 ventral root. Neurobiol (Bp). 1996;4:231–2. 9044350

[6] Pintér S, Gloviczki B, Szabó A, Márton G, Nógrádi A. Increased survival and reinnervation of cervical motoneurons by riluzole after avulsion of the C7 ventral root. J Neurotrauma. 2010;27:2273–82. 20939695 10.1089/neu.2010.1445 PMC3136727

[7] Zhang W, Xiao D, Mao Q, Xia H. Role of neuroinflammation in neurodegeneration development. Signal Transduct Target Ther. 2023;8:267. 37433768 10.1038/s41392-023-01486-5 PMC10336149

[8] Ribeiro TB, Duarte AS, Longhini AL, Pradella F, Farias AS, Luzo AC, Oliveira AL. Olalla Saad ST: neuroprotection and Immunomodulation by xenografted human mesenchymal stem cells following spinal cord ventral root avulsion. Sci Rep. 2015;5:16167. 26548646 10.1038/srep16167 PMC4637826

[9] Spejo AB, Carvalho JL, Goes AM, Oliveira AL. Neuroprotective effects of mesenchymal stem cells on spinal motoneurons following ventral root axotomy: synapse stability and axonal regeneration. Neuroscience. 2013;250:715–32. 23896572 10.1016/j.neuroscience.2013.07.043

[10] Schlett K, Herberth B, Madarász E. In vitro pattern formation during neurogenesis in neuroectodermal progenitor cells immortalized by p53-deficiency. Int J Dev Neurosci. 1997;15:795–804. 9402230 10.1016/s0736-5748(97)00015-4

[11] Pajer K, Bellák T, Redl H, Nógrádi A. Neuroectodermal stem cells grafted into the injured spinal cord induce both axonal regeneration and morphological restoration via multiple mechanisms. J Neurotrauma. 2019;36:2977–90. 31111776 10.1089/neu.2018.6332 PMC6791485

[12] Kang GH, Yan BC, Cho GS, Kim WK, Lee CH, Cho JH, Kim M, Kang IJ, Won MH, Lee JC. Neuroprotective effect of Fucoidin on lipopolysaccharide accelerated cerebral ischemic injury through Inhibition of cytokine expression and neutrophil infiltration. J Neurol Sci. 2012;318:25–30. 22560605 10.1016/j.jns.2012.04.013

[13] Gál L, Bellák T, Marton A, Fekécs Z, Weissman D, Török D, Biju R, Vizler C, Kristóf R, Beattie MB, et al. Restoration of motor function through delayed intraspinal delivery of human IL-10-Encoding Nucleoside-Modified mRNA after spinal cord injury. Res (Wash D C). 2023;6:0056. 10.34133/research.0056 PMC10013810 36930811

[14] Arevalo-Martin A, Garcia-Ovejero D, Sierra-Palomares Y, Paniagua-Torija B, Gonzalez-Gil I, Ortega-Gutierrez S, Molina-Holgado E. Early endogenous activation of CB1 and CB2 receptors after spinal cord injury is a protective response involved in spontaneous recovery. PLoS ONE. 2012;7:e49057. 23152849 10.1371/journal.pone.0049057 PMC3496738

[15] Török DG, Fekécs Z, Pajer K, Pintér S, Nógrádi A. The use of a detailed video-based locomotor pattern analysis system to assess the functional reinnervation of denervated Hind limb muscles. J Neurosci Methods. 2022;365:109398. 34728254 10.1016/j.jneumeth.2021.109398

[16] Sorokin SP, Hoyt RF. Macrophage development: I. Rationale for using Griffonia simplicifolia isolectin B4 as a marker for the line. Anat Rec. 1992;232:520–6. 1372795 10.1002/ar.1092320409

[17] Cizkova D, Novotna I, Slovinska L, Vanicky I, Jergova S, Rosocha J, Radonak J. Repetitive intrathecal catheter delivery of bone marrow mesenchymal stromal cells improves functional recovery in a rat model of contusive spinal cord injury. J Neurotrauma. 2011;28:1951–61. 20822464 10.1089/neu.2010.1413

[18] Krupa P, Vackova I, Ruzicka J, Zaviskova K, Dubisova J, Koci Z, Turnovcova K, Urdzikova LM, Kubinova S, Rehak S, Jendelova P. The Effect of Human Mesenchymal Stem Cells Derived from Wharton’s Jelly in Spinal Cord Injury Treatment Is Dose-Dependent and Can Be Facilitated by Repeated Application. Int J Mol Sci 2018, 19. 10.3390/ijms19051503 PMC5983761 29772841

[19] Walcheck B, White M, Kurk S, Kishimoto TK, Jutila MA. Characterization of the bovine peripheral lymph node homing receptor: a lectin cell adhesion molecule (LECAM). Eur J Immunol. 1992;22:469–76. 1371468 10.1002/eji.1830220227

[20] Butcher EC. Leukocyte-endothelial cell recognition: three (or more) steps to specificity and diversity. Cell. 1991;67:1033–6. 1760836 10.1016/0092-8674(91)90279-8

[21] Lasky LA. Selectins: interpreters of cell-specific carbohydrate information during inflammation. Science. 1992;258:964–9. 1439808 10.1126/science.1439808

[22] Li X, Steeber DA, Tang ML, Farrar MA, Perlmutter RM, Tedder TF. Regulation of L-selectin-mediated rolling through receptor dimerization. J Exp Med. 1998;188:1385–90. 9763619 10.1084/jem.188.7.1385 PMC2212497

[23] Wang Q, Tang XN, Yenari MA. The inflammatory response in stroke. J Neuroimmunol. 2007;184:53–68. 17188755 10.1016/j.jneuroim.2006.11.014 PMC1868538

[24] Ley K, Laudanna C, Cybulsky MI, Nourshargh S. Getting to the site of inflammation: the leukocyte adhesion cascade updated. Nat Rev Immunol. 2007;7:678–89. 17717539 10.1038/nri2156

[25] Zhang XW, Liu Q, Thorlacius H. Inhibition of selectin function and leukocyte rolling protects against dextran sodium sulfate-induced murine colitis. Scand J Gastroenterol. 2001;36:270–5. 11305514 10.1080/003655201750074555

[26] Davies JM, Radford KJ, Begun J, Levesque JP, Winkler IG. Adhesion to E-selectin primes macrophages for activation through AKT and mTOR. Immunol Cell Biol. 2021;99:622–39. 33565143 10.1111/imcb.12447

[27] Moezzi D, Dong Y, Jain RW, Lozinski BM, Ghorbani S, D’Mello C, Wee Yong V. Expression of antioxidant enzymes in lesions of multiple sclerosis and its models. Sci Rep. 2022;12:12761. 35882921 10.1038/s41598-022-16840-w PMC9325863

[28] Molnár K, Nógrádi B, Kristóf R, Mészáros Á, Pajer K, Siklós L, Nógrádi A, Wilhelm I, Krizbai IA. Motoneuronal inflammasome activation triggers excessive neuroinflammation and impedes regeneration after sciatic nerve injury. J Neuroinflammation. 2022;19:68. 35305649 10.1186/s12974-022-02427-9 PMC8934511

